# Fire and Brimstone: Molecular Interactions between Sulfur and Glucosinolate Biosynthesis in Model and Crop Brassicaceae

**DOI:** 10.3389/fpls.2016.01735

**Published:** 2016-11-21

**Authors:** Priyakshee Borpatragohain, Terry J. Rose, Graham J. King

**Affiliations:** ^1^Southern Cross Plant Science, Southern Cross University, LismoreNSW, Australia; ^2^Southern Cross GeoScience, Southern Cross University, LismoreNSW, Australia

**Keywords:** allyl-isothiocyanate, mustard, internal signaling, sinigrin, sulfate, canola, *Brassicaceae*

## Abstract

Glucosinolates (GSLs) represent one of the most widely studied classes of plant secondary metabolite, and have a wide range of biological activities. Their unique properties also affect livestock and human health, and have been harnessed for food and other end-uses. Since GSLs are sulfur (S)-rich there are many lines of evidence suggesting that plant S status plays a key role in determining plant GSL content. However, there is still a need to establish a detailed knowledge of the distribution and remobilization of S and GSLs throughout the development of *Brassica* crops, and to represent this in terms of primary and secondary sources and sinks. The increased genome complexity, gene duplication and divergence within brassicas, together with their ontogenetic plasticity during crop development, appear to have a marked effect on the regulation of S and GSLs. Here, we review the current understanding of inorganic S (sulfate) assimilation into organic S forms, including GSLs and their precursors, the intracellular and inter-organ transport of inorganic and organic S forms, and the accumulation of GSLs in specific tissues. We present this in the context of overlapping sources and sinks, transport processes, signaling molecules and their associated molecular interactions. Our analysis builds on recent insights into the molecular regulation of sulfate uptake and transport by different transporters, transcription factors and miRNAs, and the role that these may play in GSL biosynthesis. We develop a provisional model describing the key processes that could be targeted in crop breeding programs focused on modifying GSL content.

## Introduction

Glucosinolates are sulfur-rich, nitrogen-containing secondary metabolites with a wide range of biological activities. Their unique properties also affect livestock and human health, and have been harnessed for food and other end-uses. Almost, 200 different GSLs have been identified to date and are predominantly reported in the order *Brassicales* (*Capparales*) (reviewed in Fahey et al., 2001; Clarke, 2010). However, some GSLs have also been identified in non-cruciferous dicotyledonous angiosperms (Rodman et al., 1996). The evolutionary role of these molecules appears to have arisen from the selective advantage conferred due to their insect anti-feedant or attraction properties (reviewed in Bones and Rossiter, 1996; Wittstock and Burow, 2010), nematicidal (Lazzeri et al., 1993; Buskov et al., 2002) and fungal disease suppression (Mari et al., 2002; Tunc et al., 2007; Bednarek et al., 2009; Hacquard et al., 2016; Hiruma et al., 2016) as well as herbicidal properties (Brown and Morra, 1995; Vaughn et al., 2006).

Until about 40 years ago oilseed crop cultivars of *Brassica napus* and *Brassica rapa* possessed high levels of GSLs, ranging from 100 to 180 μmol/g in defatted seed meal, which hindered the use of the protein-rich rapeseed meal because of its anti-nutritional effects on livestock (reviewed in Griffiths et al., 1998). Following discovery of a low GSL-containing Polish spring rape cultivar, Bronowski (Kondra and Stefansson, 1970), the production and consumption of *B. napus* increased rapidly and it now represents 15% of total global vegetable oil (USDA, 2016). The name ‘canola’ was first used by the Rapeseed Association of Canada, but it is now a general name for any rapeseed or oilseed rape cultivars with ‘double low’ levels of erucic acid (<2%) and GSL (<30 μmol/g in oil-free seed meal) (Canola Council, 2016).

However, GSLs and their derivatives also have a wide range of positive attributes in the context of food production, human nutrition and other end-uses such as in biofumigation. The unique S-based properties of GSLs impart the distinctive flavors and pungency of cruciferous vegetables and oils, beneficial effects on human health and anti-carcinogenic properties (Hayes et al., 2008). For example, sulforaphane, a derivative of glucoraphanin and mostly found in broccoli (*B. oleracea* var. italica), can act as a preventive agent for certain types of cancer (Brooks et al., 2001). Phenyl group-containing isothiocyanates of some GSLs and indole-3-carbinol derivatives of glucobrassicin are also reported to suppress the growth of mammalian carcinogenic cells (Hecht, 2000; Nakajima et al., 2001; Choi et al., 2010).

The volatile mustard oil ally-isothiocyanate (AITC), produced from defatted *Brassica juncea* seed meal, is used as a food flavoring agent, and as a natural preservative because it has been shown to inhibit the growth of certain fungi and bacteria (Mari et al., 2002; Rhee et al., 2003; Bednarek et al., 2009). Isothiocyanates (ITCs) can also be used as biofumigants for soil-borne pests (Brown and Morra, 2005; Bellostas et al., 2007a). Because of the desirability of many key GSLs and their hydrolyzed products – especially isothiocyanate – there has been strong interest in understanding the genetic regulation of GSLs in order to manipulate their content in crop plants through plant breeding (reviewed in Mithen, 2001).

**FIGURE 1:**
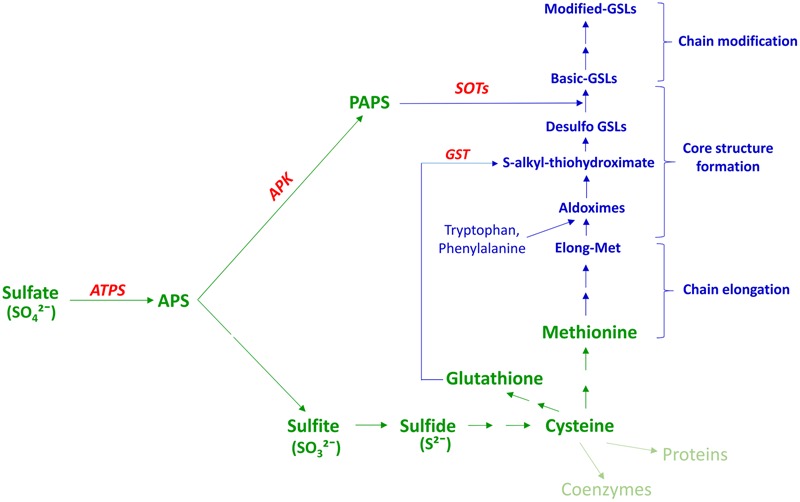
**Association between plant S assimilation and GSL biosynthesis.** Modified from Sonderby et al. (2010); Mugford et al. (2011) PAPS, 3′-phosphoadenosine 5′-phosphosulfate; APS, adenosine 5′-phosphosulphate; ATPS, ATP sulfurylase; APK, APS kinase; GSL, glucosinolate; GST, glutathione -*S*-transferase; SOT, sulfotransferase. Text in green indicates the S-assimilation process, and text in blue GSL biosynthesis. Enzymes are shown in red.

While total GSL content in most *Brassica* species is typically less than 1% of the total plant dry weight (Fieldsend and Milford, 1994; Blake-Kalff et al., 1998; Bellostas et al., 2007a), a substantial amount of S can be sequestered within the GSLs – between 1.7 and 8.0% of total plant S content (reviewed in Falk et al., 2007). Many of the predominant methionine (Met) derived GSLs have three S atoms in their structure (reviewed in Falk et al., 2007; Frerigmann and Gigolashvili, 2014b), one of which derives from the amino acid precursor. *Arabidopsis* phytoalexinine (pad2) mutant and adenosine-5′-phosphosulfate kinase (apk1apk3apk4) mutant shown that the remaining two S atoms, in common with other GSLs, originate from glutathione (GSH) (Geu-Flores et al., 2011) and 3′-phosphoadenosine 5′-phosphosulfate (PAPS) produced in the S-assimilation pathway (Mugford et al., 2009; **Figure 1**). As such, GSL-containing species of the *Brassicaceae* such as oilseed rape have a higher S requirement than non-GSL-containing species (Klessa and Sinclair, 1989). Under S deficient conditions, synthesis of S-containing amino acids is restricted, and this can reduce the photosynthetic activity of the plant leading to reduced plant growth (Ahmad and Abdin, 2000). Moreover, there is evidence that application of relatively higher rates of S fertilizer in *B*. *rapa* can lead to preferential accumulation of aliphatic and aromatic rather than indole GSLs (Chen et al., 2006). In most field and greenhouse trials, as well as in experiments carried out in artificial media, GSL content is sensitive to increased S availability levels, with up to 10-fold increases reported in some cases (reviewed in Walker and Booth, 2003; Falk et al., 2007; Supplementary Table S1). However, few of these studies report detailed dose response curves, and ultimately any GSL response to S fertilization will depend on the existing native soil S status and will be site and season dependent.

In recent years, plant S metabolism has been well-reviewed in terms of products, substrates and associated enzyme (Takahashi et al., 2011; Calderwood et al., 2014) as subsequently in terms of transport (Gigolashvili and Kopriva, 2014) and molecular regulation of the key processes of S-assimilation (Koprivova and Kopriva, 2014, 2016). However, our understanding of the molecular regulation of GSL biosynthesis in relation to S availability is less clear. Here, we review the current understanding of inorganic S (sulfate) assimilation into organic S forms, including GSLs and their precursors, the intracellular and inter-organ transport of inorganic and organic S forms, and the accumulation of GSLs in specific tissues. We present this in the context of overlapping sources and sinks, transport processes, signaling molecules and their associated molecular interactions. We refer to sources as the specific organs and tissues associated with S assimilation and GSL biosynthesis, and sinks as those associated with storage or use of assimilates. Our analysis builds on recent insights into the molecular regulation of sulfate uptake and transport by different transporters, transcription factors (TFs) and miRNAs, and the role that these may play in GSL biosynthesis. We develop a provisional model describing the key processes that may be manipulated in breeding programs focused on modifying GSL content.

## Chemistry and Biosynthesis of GSLs

**FIGURE 2:**
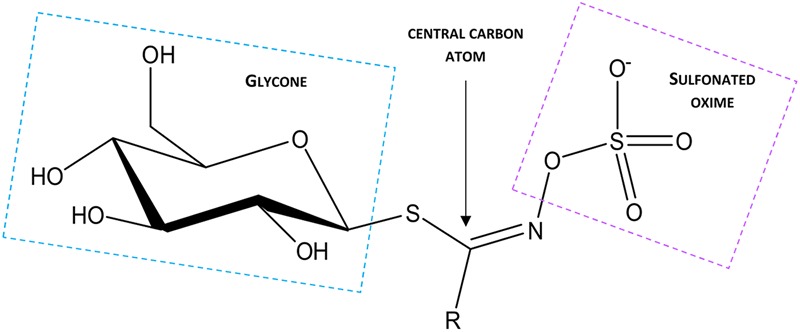
**Structure of the canonical GSL molecule, where R denotes the variable side chain**.

Glucosinolates have been known for 1000s of years as pungency of mustard oil and bitter flavor of cruciferous vegetables. The basic structures of singrin and sinalbin were first elucidated in Ettlinger and Lundeen (1956). Subsequently, the underlying process of GSL hydrolysis was found to be responsible for the specific flavor properties within the brassicaceae (Kjaer, 1976; Tookey et al., 1980; Fenwick et al., 1983). GSLs are anionic, with the core structure of each molecule having a centrally localized carbon atom which is linked to a glycone group via a thioglucoside bond which originates from GSH. In addition, the central carbon atom links to a sulfonated oxime via an N bond, and is also linked to an amino acid-derived variable side chain (R group) (reviewed in Fahey et al., 2001; **Figure 2**). In some cases, other components are also attached to the glycone moiety or oxygen (O), S, or N atoms of the R group (reviewed in Agerbirk and Olsen, 2012). The background to the chemistry and molecular regulation of glucosinolates has been addressed in many extensive reviews (Halkier and Gershenzon, 2006; Sonderby et al., 2010; Agerbirk and Olsen, 2012; Baskar et al., 2012; Ishida et al., 2014). It was the detailed characterization of GSLs and associated pathways in *Arabidopsis* that opened the way for a molecular understanding of the genes encoding specific enzymes associated with each synthetic and modification step.

Almost, all GSL-producing plants also possess a β-thioglucoside glycohydrolase enzyme known as myrosinase (reviewed in Rask et al., 2000; Bellostas et al., 2007b). Upon tissue damage or insect herbivory, endogenous myrosinase and vacuolar GSLs come in contact with each other, resulting in hydrolyzation of the GSL to form a variety of compounds such as isothiocyanates, epithionitriles, nitriles, thiocyanates, and oxazolidines, depending on the structure of the substrate GSL, pH and on the presence of metal ions, additional proteins and cofactors (reviewed in Bones and Rossiter, 1996, 2006; Halkier and Gershenzon, 2006; Kissen and Bones, 2009).

Glucosinolates are categorized into three major types according to their precursor amino acids. In different tissues of *Brassica*, aliphatic GSLs account for 70–97% of the total GSL content. This has been reported in leaves of *B. oleracea* (Cartea et al., 2008), leaves and stems of *B. napus* (Cleemput and Becker, 2012), leaves and seeds of *B. juncea* (Shilpa et al., 2012; Othmane, 2015) and sprouts and mature leaves of *B. rapa* (Wiesner et al., 2013). Aliphatic GSLs are mostly derived from Met, with a few from leucine, isoleucine, alanine, and valine. In *Arabidopsis*, aliphatic and indole GSLs are the most abundant in different tissues (Kliebenstein et al., 2001; Brown et al., 2003). However, aromatic GSLs are the minor components in both *Brassica* and *Arabidopsis*. The indole GSLs are derived from tryptophan (Trp), whereas aromatic GSLs are derived from phenylalanine (Phe) and tyrosine (reviewed in Fahey et al., 2001; Mithen, 2001; Halkier and Gershenzon, 2006).

There are three independent enzyme-mediated phases of GSLs biosynthesis, which have been well-characterized in *Arabidopsis thaliana*: amino acid side-chain elongation, core GSL structure formation, and side chain modification, the first and last of which are dependent upon substrate specificity (reviewed in Grubb and Abel, 2006; Halkier and Gershenzon, 2006; Sonderby et al., 2010; Baskar et al., 2012; **Figure 1**). Although, aliphatic and aromatic GSLs usually undergo side-chain elongation, this process has not been reported in the case of indole GSLs (Textor et al., 2004). The chain elongation pathway is mediated by branched chain aminotransferases (BCATs), methyl thioalkyl malate synthases (MAMs), isopropyl malate isomerase (IPMI) and isopropyl malate dehydrogenase (IPM-DH) (Schuster et al., 2006; Knill et al., 2008; Gigolashvili et al., 2009b; Sawada et al., 2009a,b; Zhang et al., 2015a). The resulting chain-elongated amino acid can either enter into the core GSL structure formation phase, or proceed through additional chain elongation cycles to add further methylene groups (reviewed in Halkier and Gershenzon, 2006). The GSL core structure formation phase involves enzymes of the CYP79 (Hull et al., 2000; Mikkelsen et al., 2000; Hansen et al., 2001; Chen et al., 2003), CYP83 (Bak and Feyereisen, 2001; Hemm et al., 2003; Naur et al., 2003), UGT74 (Grubb et al., 2004, 2014; Gachon et al., 2005) families, C-S-lyases (Mikkelsen et al., 2004) and sulfotransferases (SOTs or STs) (Piotrowski et al., 2004). These enzymes facilitate the biosynthesis of basic GSL structures from elongated and non-elongated amino acids. Notably, GSH produced in the S-assimilation process is considered as the preferred S donor for synthesis of *S*-alkyl-thiohydroximate via GSH-*S*-transferase (GST) in the core structure development phase (Schlaeppi et al., 2008; Dixon et al., 2010; Geu-Flores et al., 2011; **Figure 1**). On the other hand, basic GSLs are produced only after the sulfation of desulfo-GSLs with a PAPS sulfate donor, catalyzed by SOTs (**Figure 1**). As cysteine (Cys) is the precursor for GSH as well as Met, Cys and PAPS can be considered as the major connection between GSL biosynthesis and the S assimilation process (**Figures 1, 5**, and **6**). After synthesis, these basic GSL structures are subjected to a range of secondary side chain modification and transformation pathways involving enzymes such as flavin mono oxygenase (FMOOXs) (Hansen et al., 2007; Li et al., 2008), GSL-AOPs (Mithen et al., 1995), GSL-OH (Hansen et al., 2008) and CYP81Fs (Pfalz et al., 2009, 2011) to generate different types of GSL structures (reviewed in Fahey et al., 2001; Clarke, 2010; **Figure 1**).

### Candidate Genes and Signaling Molecules Involved in GSL Biosynthesis

The genes encoding enzymes associated with each of the known steps of GSL synthesis have been characterized in *Arabidopsis* (reviewed in Sonderby et al., 2010). Amongst the enzymes of chain elongation reaction of GSL biosynthesis, the genetic loci responsible for MAMs have been resolved within the *Arabidopsis* genome (Kroymann et al., 2001; Textor et al., 2007; Sawada et al., 2009b; Redovnikovic et al., 2012), and more recently in different *Brassica* species (Gao et al., 2006; Liu et al., 2011; Sotelo et al., 2014; Zhang et al., 2015a). An early confirmation of functional conservation within the *Brassicaceae* came from cloning *B. oleracea* homologs of *AtGSL-Elong/AtMAM* (*BoGSL-Elong*) and of *AtGS-ALK* (*BoGSL-ALK*+), with confirmation of their involvement in GSL biosynthesis by functional analysis (Li and Quiros, 2002, 2003), In particular, the function of the *BoGSL-ALK* gene was demonstrated by transgenic gene complementation of *A. thaliana* ecotype Columbia, in which the corresponding allele for *GSL-ALK* is non-functional. In addition, the *BoGSL-PRO* gene cloned from *B. oleracea* was found to be involved in the side chain elongation phase of aliphatic GSL synthesis (Li et al., 2001). Comparative genomic analysis has been extended, firstly via physical mapping, with *B. oleracea* BAC clones containing orthologs of the *AOP* (B21H13) and *MAM* (B21F5) families providing insights into gene conservation and divergence between *A. thaliana* and *B. oleracea* (Gao et al., 2004, 2006). Putative orthologs of five major genes in the GSL biosynthetic pathway (*BraGSL-ELONGa, BraGSL-ALKa, BraCYP83B1, BraSUR1a*, and *BraST5a*) were cloned from both cDNA and genomic DNA of different subspecies of *B. rapa* (Yang et al., 2010), with expression studies substantiating their involvement in GSL biosynthesis. Likewise, putative orthologs of *Arabidopsis CYP83A1* and *CYP83B1* isolated from *B. rapa*, including *BrCYP83A1* and *BrCYP83B1* were characterized by qRT-PCR analysis (Zhu et al., 2012). Twenty one ‘metabolite’-quantitative trait loci (mQTL) have been identified for total GSL in the seeds of *B. napus* (AC genome) (Feng et al., 2012), of which nine correspond to the QTLs reported earlier in the same species (Toroser et al., 1995; Uzunova et al., 1995; Howell et al., 2003; Quijada et al., 2006). This study (Feng et al., 2012), also identified twenty-seven compositional mQTLs for aliphatic and indole GSL accumulation in leaves, of which four correspond to those previously identified in *B. rapa* (A genome) for aliphatic and indole GSL accumulation in leaves (Lou et al., 2008). A further 25 mQTL for seed aliphatic GSLs were identified on the A genome (Feng et al., 2012), of which only four are thought to have been identified previously in *B. juncea* (AB genome) (Ramchiary et al., 2007; Bisht et al., 2009). Very recently, biparental and association mapping studies in *B. juncea* (Rout et al., 2015) and *B. napus* (Qu et al., 2015) identified additional QTLs associated with GSL compositional variation and seed GSL accumulation.

**FIGURE 3:**
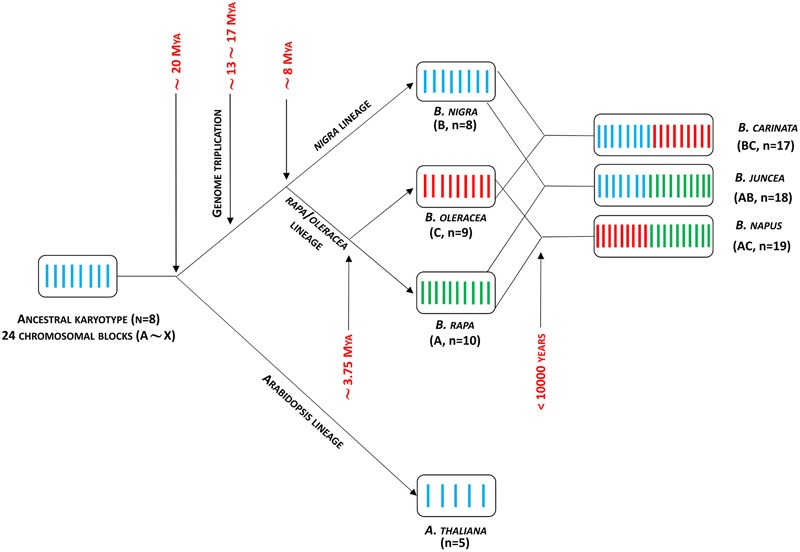
***Brassica* genome evolution model.** Adapted from Liu et al. (2014). Mya, million years ago.

Since divergence from a common ancestor over 20 million years ago (Liu et al., 2014) the genomes of the diploid crop brassicas *B. rapa* (A genome), *B. nigra* (B) and *B. oleracea* (C) have undergone duplications leading to a triplicated mesopolyploid structure (Wang X. et al., 2011), which is then duplicated in the amphidiploids *B. juncea* (AB), *B. napus* (AC), and *B. carinata* (BC) (**Figure 3**). This has led to considerable diversity resulting from gene duplication and divergence, with opportunities for neo-functionalization in terms of temporal and spatial gene regulation. The divergence of the diploid *Brassica* genomes over the past 5–10 million years has resulted in a distinct pattern of aliphatic GSL composition associated with each of the A, B, or C genomes. Thus, GSLs with three carbon side chains derived from a single elongation reaction are found in *B. nigra*, whereas *B. oleracea* contains three or four carbon side chains, and *B. rapa* contains four or five carbon side chain GSLs. The three amphidiploid species each have a GSL composition corresponding to their respective diploid genome composition (reviewed in Ishida et al., 2014). The greater opportunity for gene divergence and specialization driven by the complex genome structure of brassicas also appears to provide these crop species with considerable ontogenetic plasticity during plant development, and selection of a wide range of morphotypes.

Recently, 82 mQTLs associated with GSL compositional profiles were identified in *B. oleracea*, which corresponded by collinearity with orthologous *Arabidopsis* GSL biosynthesis genes (Sotelo et al., 2014). A separate study has indicated that three paralagous genes encoding AOP2 protein involved in aliphatic GSL biosynthesis, are differentially expressed in *B. rapa* (Zhang et al., 2015b). Although, there is evidence of a reduction in GSL biosynthesis and related genes due to recent breeding selection in canola-type *B. napus* (Chalhoub et al., 2014), the ability of these polyploid genomes to carry multiple copies of biosynthetic and regulatory genes at upward of six or more paralogous loci provides an opportunity for far greater complexity. The collinearity of GSL biosynthesis genes between *Arabidopsis* and *Brassica* genomes and their proliferation in *Brassica* has been established through comparative whole-genome analysis in *B. rapa* (Wang H. et al., 2011), as well as in *B. oleracea* (Liu et al., 2014), *B. napus* (Chalhoub et al., 2014), and *B. juncea* (Yang et al., 2016) (Supplementary Table S2).

A complex network of TFs belonging to the R2R3-MYB family regulate the biosynthesis of GSLs in *Arabidopsis*. These are distinct from those involved in generic S metabolism and transport, which we discuss later. MYB28, MYB76, and MYB29 TFs influence the expression of MAMs, CYP79F1/F2, CYP83A1, SUR1, UGT74B1, and SOT17/18 genes involved in aliphatic GSL biosynthesis (Gigolashvili et al., 2007, 2008, Hirai et al., 2007; Sønderby et al., 2007). A complementary set of TFs including MYB51, MYB122, and MYB34 appear to regulate indole GSL biosynthesis via CYP79B2/B3, CYP83B1, SUR1, UGT74B1, and SOT16 genes (Yatusevich et al., 2010; Frerigmann and Gigolashvili, 2014a). Four differentially expressed *At*MYB28 homologs *in B. juncea* can act as major transcriptional regulators of aliphatic GSLs accumulation (Augustine et al., 2013a). Moreover, targeted gene silencing of these MYBs suggests that *Bj*MYB28 would be a potential candidate for developing low GSL lines in *B. juncea* (Augustine et al., 2013b). However, recent functional analysis in transgenic *B. rapa* of three orthologous copies of *At*MYB28 revealed that *Br*MYB28 genes are responsible not only for aliphatic, but also for indole and aromatic GSLs biosynthesis, where they specifically act as positive regulators of the *Br*GSL-OH gene and negatively regulate the *Br*AOP2 genes (Seo et al., 2016).

Moreover, evidence from yeast-two-hybrid (Y2H) screening (Frerigmann et al., 2014) and mutant analysis in *Arabidopsis* (Schweizer et al., 2013) has revealed that four members of another group of TFs belonging to the bHLH subgroup IIIe, also known as MYC-bHLH TFs, regulate GSL biosynthesis in cooperation with MYB TFs (reviewed in Frerigmann, 2016). MYC-bHLHs also act as key signaling components of the jasmonic acid pathway (Fernandez-Calvo et al., 2011). Besides these six MYBs and four MYCs, two new MYBs (MYB115 and MYB118) have recently been identified which are responsible for the regulation of aliphatic GSLs in *Arabidopsis* (Zhang Y. et al., 2015). A further recent study based on an *Arabidopsis* mutant of a small heme-binding protein, known as Cytochrome b5 (CB5C), showed that it can influence GSL biosynthesis via cytochrome P450 (Vik et al., 2016). However, the mode of action of this protein is still unknown.

## Sites of GSL Synthesis and Accumulation in Plants

Within intact plants, the site of GSL synthesis is physically segregated in tissues distinct from the site of GSL storage (Chen et al., 2001), most likely to protect biologically active cells from the cytotoxic effects of GSL hydrolysis products. However, this may also be the most resource efficient means by which a centrally localized biosynthetic machinery can deliver GSLs to several sink tissues (reviewed in Kissen et al., 2009; Jørgensen et al., 2015). There is additional separation of the GSL substrates and myrosinase enzymes in a stable spatial distribution system (reviewed in Winde and Wittstock, 2011) originally known as the ‘mustard oil bomb’ (Lüthy and Matile, 1984). This has been characterized by the typical localization of GSLs in specialized S-cells of high S content, with myrosinases localized in distinct myrosin cells (Koroleva and Cramer, 2011). Additional evidence from metabolite profiling in *Arabidopsis* has suggested a separate system wherein GSLs and myrosinase can co-exist in the same cell, with GSLs localized within vacuoles and ‘atypical myrosinase (PEN2 protein)’ in endoplasmic reticulum (ER) bodies, which are derived vesicles of the ER (Moussaieff et al., 2013). The presence of similar ER bodies in *Arabidopsis*, as well as in some other members of the *Brassicales*, suggests that this distribution is a common feature within these taxa (reviewed in Nakano et al., 2014). Moreover, a recent confocal laser scanning microscopy study of *Arabidopsis pen2* mutant plants has indicated that the ‘atypical myrosinase’ has a dual-membrane targeting capability toward peroxisomes and mitochondria (Fuchs et al., 2016).

The concentration of GSLs varies between tissue types and developmental stage, with a study of 29 different plant species demonstrating that greater diversity and higher concentrations of GSLs exist in roots compared to shoots during vegetative growth (van Dam et al., 2009). In mature *Arabidopsis*, the highest concentrations of GSLs are found in seeds (2.5–3.3% of dry weight), followed by inflorescences and siliques (0.6–1.0%) and cauline leaves, roots, stems, and rosette leaves (0.3–1.0%) (Brown et al., 2003). Similar distributions of GSLs are observed in *Brassica* plants (Fahey et al., 2001; Rangkadilok et al., 2002; Bhandari et al., 2015). A GSL tissue audit in *B. napus* has suggested that changes within sink tissues are determined according to varying requirements at specific plant developmental stages (Clossais-Besnard and Larher, 1991; Porter et al., 1991).

**FIGURE 4:**
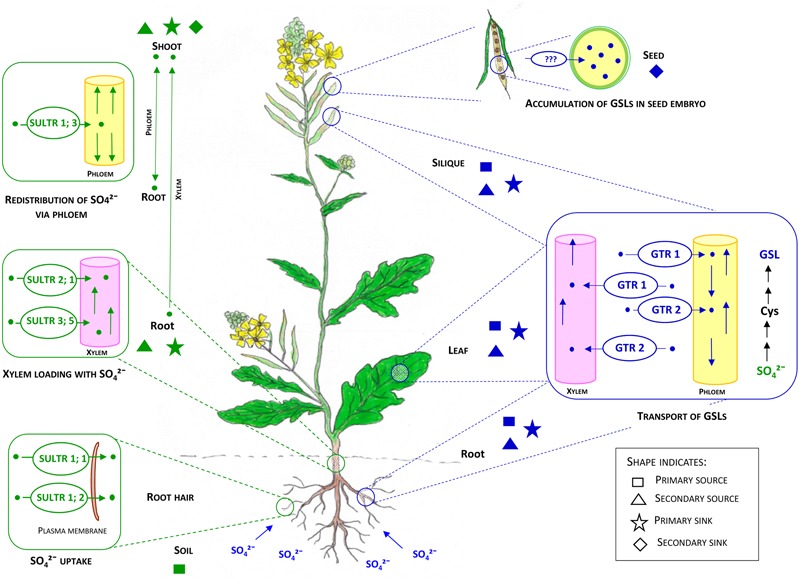
**Internal transport systems of sulfate (SO_4_^2-^) and GSLs in the context of primary and secondary sources and sinks in brassicas.** Adapted from Frerigmann and Gigolashvili (2014b); Gigolashvili and Kopriva (2014), Jørgensen et al. (2015). Soil can be considered as the primary source for S. Roots and shoots have overlapping functions as primary and secondary sources and sinks for S. Sulfate uptake by root hairs from soil is facilitated by SULTR 1; 1 and SULTR 1; 2. Root to shoot transport of sulfate is facilitated by SULTR2; 1 and SULTR3; 5 via xylem tissues. Redistribution of sulfate is likely to occur by SULTR 1; 3, while GSLs are primarily synthesized in leaves, siliques and roots. After synthesis, GSLs can undergo bidirectional transport to the same and/or other organs or tissues by GTR 1 and GTR2. There is an overlap of primary and secondary sources and sinks for GSL (leaf, root, and silique). Seed embryos can be considered as the final sink for GSLs in mature *Brassica* plants. GTR1 and GTR2 facilitate the transport process by vascular and symplastic domains. The critical barrier (e.g., the funiculus) for localization of GTRs to facilitate transport of GSLs to embryo is still unknown. Text and shapes in green indicate transport, sources and sinks related to the sulfate system whereas text and shapes in blue indicate the GSL system.

It has been suggested that siliques and rosette leaves are the primary sources for the GSLs accumulated in the seeds (secondary sink) of *B. napus* (Magrath and Mithen, 1993; Bloem et al., 2007), *B. oleracea* (Bhandari et al., 2015), *Sinapis alba* (Du and Halkier, 1998) and *Arabidopsis* (Chen et al., 2001; Ellerbrock et al., 2007) (**Figure 4**). This is supported by recent studies with mutants of *Arabidopsis* GSL transporters (Nour-Eldin et al., 2012), although confirmatory evidence for primary sources of GSLs accumulated in seed (embryo) has yet to emerge (reviewed in Jørgensen et al., 2015). Roots and trichomes are also capable of aliphatic and indole-GSL biosynthesis in *Arabidopsis* (Frerigmann et al., 2012; Andersen et al., 2013). Based on micro-grafting experiments in *Arabidopsis*, distinct source-sink relationships have been found to be associated with short- and long chained aliphatic GSLs. For long-chained aliphatic GSLs, roots appear to act as a source and rosette leaves as a sink, whereas for short chained-aliphatic GSLs, rosettes can act as both source and sink (Andersen et al., 2013; Moussaieff et al., 2013; Andersen and Halkier, 2014). This evidence has been used to suggest that there must be specific regulation of GSL distribution between different tissues within the plant (reviewed in Jørgensen et al., 2015). Moreover, evidence from transcriptomic studies of sulfate transporters (SUTRs) (Buchner et al., 2004b) suggests that primary sink tissues (e.g., leaf, silique, root) associated with S assimilation at an early stage of plant development may later act as a source for primary and secondary sinks of GSLs, such as siliques and seeds (Andersen et al., 2013). However, there remains an incomplete picture of how GSL source and sink distribution changes in relation to plant and crop developmental phases.

## Transport of Inorganic and Organic S

Sulfur is an essential macronutrient involved in numerous metabolic activities required for plant growth, especially in amino acid and protein synthesis. The primary inorganic form, sulfate, accounts for 42–94% of the total S content in different tissues of *Brassica* plants (Blake-Kalff et al., 1998; Castro et al., 2004), whereas other inorganic forms (sulfite and elemental sulfur) are much lower in concentration. Notably, in *Brassica* seeds, proteins (cruciferins and napins) and GSLs represent the major sinks of S, with each representing an approximate similar stoichiometric ratio of S relative to seed mass (Supplementary Table S3). However, this ratio is sensitive to the relative composition of cruciferins to napins, suggesting scope for manipulating GSL as an S sink.

### Transport of Sulfate

**FIGURE 5:**
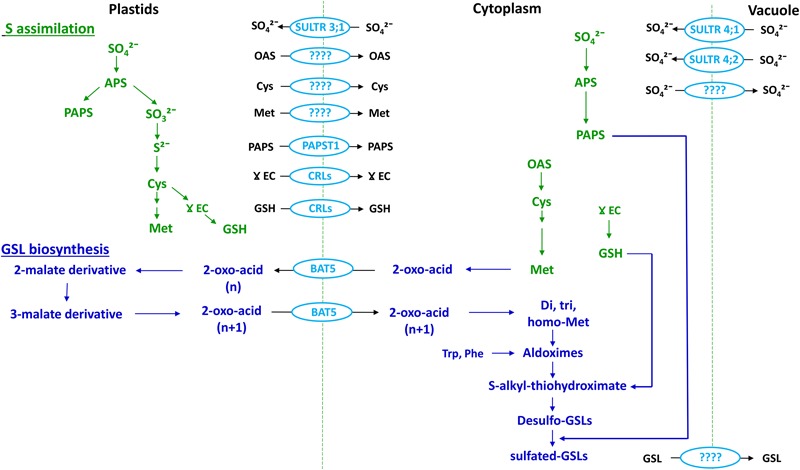
**Association between the S assimilation pathway and GSL biosynthesis (emphasizing Met-derived GSLs) within a cell.** Adapted from Gigolashvili and Kopriva (2014). OAS, *O*-acetylserine. Cellular compartments are indicated at the top of the figure. Text in green indicates S assimilation; text in dark blue indicates GSL biosynthesis and text and shapes in light blue indicate transporters.

Plants maintain a well-organized transport system to make nutrients available within the whole plant, and to distribute important compounds to specific organs. Sulfur assimilation primarily takes place in the shoot chloroplast despite roots containing all the necessary sulfate reductase enzymes (reviewed in Hawkesford, 2000; Hawkesford and De Kok, 2006). As a result, sulfate is the major form of S loaded in the xylem for transport from root to shoot, and is also the major form of stored S in the plant (Buchner et al., 2004b). Constant cytoplasmic sulfate concentrations are maintained throughout the plant system, whilst excess amounts are stored in the vacuole (Kaiser et al., 1989; Miller et al., 2001). Specialized sulfate transporters encoded by a multigene family play a key role in S-metabolite transport, and are divided into four distinct groups with different functions (Smith et al., 1995). In *Arabidopsis*, the SULTR family is well-characterized with orthologs also reported in *B. rapa, B. oleracea* and *B. juncea* (Buchner et al., 2004a; Koralewska et al., 2008; Wang X. et al., 2011; Chalhoub et al., 2014; Lancilli et al., 2014; Liu et al., 2014). In *Arabidopsis* the first group of SULTRs are high affinity transporters, amongst which SULTR1;1 and SULTR1;2 – found in the root hairs – facilitate the initial uptake of sulfate from the soil (Yoshimoto et al., 2002) while phloem-localized SULTR1;3 facilitates the redistribution of sulfate from secondary sources to primary and secondary sinks, i.e., root to shoot and *vice versa* (Yoshimoto et al., 2003; **Figure 4**). Group 2 SULTRs are mostly confined to vascular tissues to facilitate long distance transport of sulfate (Takahashi et al., 2000), while Group 3 transporters are the most diffuse form, localized in plastid membranes for import of sulfate (Cao et al., 2013; **Figures 4** and **5**). Group 4 SULTRs (SULTR4;1 and SULTR4;2) are found in the tonoplast, where they facilitate the eﬄux of sulfate from vacuoles into the cytoplasm (Kataoka et al., 2004; **Figure 5**). However, transporters responsible for sulfate influx into the vacuole are yet to be identified. It is reasonable to assume that *Brassica* SULTR homologs would have a similar or identical function as *Arabidopsis* SULTRs, due to the close phylogenic relationship of these genera (**Figure 4**). However, transcript analysis of SULTRs indicates a spatial contrast, with a pattern of abundance for group 3 SULTRs (SULTR3;2, SULTR3;3, and SULTR3;5) in *B. oleracea* root tissues, which in *Arabidopsis* are normally expressed in the leaves (Buchner et al., 2004a). Additionally, this study showed that under S deficient condition, groups 1, 2, and 4 SULTRs have been found to be up-regulated in the roots of *B. oleracea*, whereas Group 3 SULTRs are unaffected by plant S status.

### Transport Associated with S Assimilation

Following uptake by roots, sulfate is transported to shoots and assimilated by enzymatic reduction and conversion to organic S forms. Within this process, sulfate taken up from the soil is activated to adenosine 5′-phosphosulfate (APS), which is the branching point (**Figure 1**) for subsequent steps of reduction of APS to form sulfide and PAPS. Sulfide is used for synthesis of Cys and thence GSH and Met, whereas PAPS is used for sulfation of desulfo-GSLs (Mugford et al., 2011; **Figures 1** and **5**).

Cysteine, GSH, Met, and PAPS are the major S assimilates involved in GSL synthesis, with Cys the primary form synthesized in cytoplasm, plastids, and mitochondria (Lunn et al., 1990). Although, there is evidence to support the presence of numerous transport systems for Cys, specific transporters have yet to be identified (reviewed in Gigolashvili and Kopriva, 2014; **Figure 5**).

Based on transcript analysis and transient transformation experiments, the gamma (γ)-glutamylcysteine (γEC) synthase and GSH synthase enzymes of GSH synthesis have been found to be located in plastids and cytoplasm, respectively (Wachter et al., 2005). This suggests the need for export of γ-EC to cytoplasm for synthesis of GSH. The chloroquine-resistance transporter like proteins (CRLs) facilitates the transport of γ-EC as well as GSH into cytoplasm for GSL biosynthesis (Pasternak et al., 2008; **Figure 5**). GSH is one of the assimilated forms of S and there is evidence for long distance transport of GSH within the plant (Herschbach and Rennenberg, 1995). This suggest the need for identification of cell membrane located transporters in plants (reviewed in Gigolashvili and Kopriva, 2014).

Within the S assimilation process Met is formed via the mediation of cytosolic Met synthase, and is the primary precursor for aliphatic-GSLs (Schuster et al., 2006; **Figure 5**). Initially, it had been thought that the synthesis of Met required homocysteine to be transported from plastids to the cytoplasm, as there was no evidence of a Met synthase enzyme in the plastids (Eichel et al., 1995). However, following the identification of plastid-localized Met synthase, it was concluded that plastids are indeed capable of *de novo* synthesis of Met (Ravanel et al., 2004), and so a pool of Met could be sequestered within plastids that is not available for synthesis of aliphatic GSLs (**Figure 5**). So far no specific amino acid transporters have been identified in plants to facilitate Met transport between organelles or cells.

The major portion of PAPS is synthesized in chloroplasts, whereas the sulfation reaction of GSLs takes place in the cytoplasm (Mugford et al., 2009, 2010; **Figure 5**). Notably, chloroplasts contain the SAL1 phosphatase enzyme, which can hydrolyze PAP (Estavillo et al., 2011). Therefore, there must be a plastid-localized transporter to deliver PAPS in to the cytoplasm (**Figure 5**). In *Arabidopsis*, apart from its primary function, the transporter known as thylakoid ADP/ATP carrier (TAAC) has an additional function to transport PAPS across the plastid membrane (Thuswaldner et al., 2007; Gigolashvili et al., 2012). More recently, the TAAC transporter has been renamed as PAPS transporter 1 (PAPST1) (Gigolashvili et al., 2012). However, knockout mutants of *Arabidopsis* PAPST1 contain 30–50% of aliphatic GSLs and have an unaltered levels of indole GSLs, which indicates the existence of an additional transporter for PAPS in the plastid membrane (Mugford et al., 2009). It should be recognized that all these studies have been carried out solely in *Arabidopsis*, whereas detailed investigation is required in crop brassicas in order to understand the complexity of transport system associated with S assimilation in field situations.

### Transport of GSLs

Biosynthesis of GSLs is compartmentalized within plastids and the cytoplasm, where Met-derived GSLs undergo a chain elongation process in the chloroplast, prior to the synthesis of the core structure in the cytoplasm (reviewed in Halkier and Gershenzon, 2006; Sonderby et al., 2010). Transcriptome co-expression analysis of *Arabidopsis* knock out mutants suggested that chloroplast-localized bile acid transporter 5 (BAT5) can facilitate the import of Met-derived 2-oxo acid into the chloroplast, and can also export chain elongated 2-oxo-acid to the cytoplasm for synthesis of the GSL core structure (Gigolashvili et al., 2009a; Sawada et al., 2009a; **Figure 5**). A feeding experiment of the *Atbat5* knock-out mutant with Met-derived 2-keto acids and different chain length amino acids confirmed that the BAT5 transporter has specific affinity toward only 2-keto acids (Gigolashvili et al., 2009a). After the sulfated-GSLs are formed in the cytoplasm, they need to be transported either to vacuoles or S -cells for storage. The transporter(s) responsible for influx of GSLs into the vacuole for storage is still unknown.

Proteomic analysis has indicated the absence of GSL biosynthesis machinery within the cytoplasm of S-cells, which is consistent with the need for transporters to account for the accumulation of GSLs in these cells (Koroleva and Cramer, 2011). It has been confirmed that only sulfated GSLs can be transported into the developing seeds, rather than desulfo-GSLs (Mugford et al., 2009). This strongly indicates the need for long distance transport of GSLs from primary and secondary source (leaf, root, and silique) to sink (leaf, root, silique, and embryo) tissue. The recent identification of nitrate/peptide group GSL transporters AtNPF2.10 (AtGTR1) and AtNPF2.11 (AtGTR2) in *Arabidopsis* has contributed toward an understanding of the likely transport mechanisms for GSL distribution (Nour-Eldin et al., 2012). These transporters are responsible for long distance transport of short and long-chained aliphatic GSLs from primary and secondary source tissues (root, shoot, silique) to primary and secondary sinks (leaves, embryo), by facilitating long distance transport through phloem and xylem tissues (Andersen et al., 2013; Moussaieff et al., 2013; Andersen and Halkier, 2014). However, the ability of GTRs to transport GSLs via symplastic domains suggests that they also appear to be essential for maintaining the distribution of GSLs within roots and leaves (Nour-Eldin et al., 2012). Micro-grafting experiments in *Arabidopsis* have suggested the existence of a specific-transporter in addition to GTR1 and 2 that is able to facilitate the transport of indole-GSLs between rosette leaves and roots (Andersen et al., 2013). It is reasonable to expect that these components within the *Arabidopsis* sulfate and GSL transport systems are likely to be relevant in the larger and more complex crop brassicas, but confirmation of specific roles requires further investigation (**Figure 4**). To date, there appear to be no reports of specific transporters from functional studies in brassicas. However, these transporters may be useful for engineering novel insect resistance, as a recent study has demonstrated that leaves of *Arabidopsis* mutants of *gtr1gtr2* contributes to reducing the fitness of green peach aphid (*Myzus persicae*) due to reduced availability of GSL in phloem sap, and consequent increased GSL in the tissues surrounding the phloem (Madsen et al., 2015).

## Molecular Cross-Talk Between S Availability and GSL Biosynthesis

**FIGURE 6:**
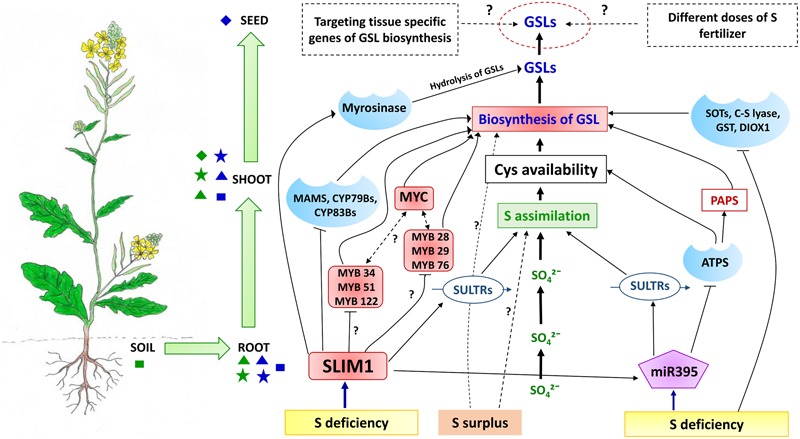
**A conceptual model how soil S status affects GSL accumulation in *Brassica* crops.** Under S deficiency, the SLIM1 TF can down regulate the expression of key GSL biosynthesis enzymes (MAMS, CYP79Bs, and CYP83Bs) and MYB TFs (Lewandowska and Sirko, 2008; Frerigmann and Gigolashvili, 2014b). Further investigation needed to understand the complete picture of regulation of MYBs targeting indole GSL biosynthesis (MYB34, MYB51, MYB 122) and aliphatic GSL synthesis (MYB28, MYB 29, MYB76) by SLIM1 under different S conditions. In contrast, under S deficient conditions SLIM1 can also upregulate the expression of SULTRs and miR395, as well as the GSL hydrolysis enzyme (myrosinase) (Takahashi et al., 2011). In addition, upregulation of miR395 via SLIM1 under S deficiency can increase the expression of SULTRs and lead to enhanced uptake of sulfate from soil. SLIM1 can also down-regulate the expression of ATPS in the first step of S assimilation (Liang et al., 2010) which can affect the availability of Cys and PAPS for GSL biosynthesis. Sulfur deficiency has been suggested as a negative regulator of SOTs, C-S lyases, GST and AOPs enzymes of the GSL biosynthesis pathway (Nikiforova et al., 2005), although additional signaling molecules may be involved in this regulation. Moreover, we would expect the presence of additional signaling molecules and TFs, which can regulate both systems (S assimilation and GSL biosynthesis) under S deficiency or varying S levels of the primary source (soil). As seeds are the ultimate sink for GSLs, optimizing the external application of S fertilizer and identifying the role of tissue-specific genes of GSL biosynthesis under different S levels would provide scope for manipulation of seed GSL levels in brassicas. Primary and secondary sources and sinks for S and GSLs are shown as indicated as in **Figure 4**. Shapes in green indicate sources and sinks related to the sulfate system whereas shapes in blue indicate the GSL system. Rectangular shapes indicate primary sources, triangles indicate secondary sources whereas stars indicate primary sinks and diamonds indicate secondary sinks.

The recent identification of feedback mechanisms between specific transporters, signaling molecules and TFs in *Arabidopsis* is leading to a better understanding of the molecular interactions that may mediate between S status and GSL biosynthesis. Metabolomic and transcriptomic studies have demonstrated that under S limitation, reduction of GSL levels (Hirai et al., 2003; Maruyama-Nakashita et al., 2003; Nikiforova et al., 2003) and activation of sulfate uptake and assimilation (Hirai et al., 2003) may occur simultaneously. So far, Sulfur Limitation 1 (SLIM1) has been reported to be the key TF in *Arabidopsis* which regulates the genes involved in sulfate uptake and S assimilation. Under S-deficient conditions, SLIM1 appears to affect both sulfate uptake and GSL biosynthesis. Transcriptome profile of *slim1 Arabidopsis* mutants clearly demonstrated that under S limitation, the expression of genes encoding SULTRs from group 1 (SULTR1;1 and SULTR1;2: for sulfate uptake by roots), group 3 (SULTR3;4; for import of sulfate) and group 4 (SULTR4;2: for release of sulfate from vacuole to cytoplasm in roots) are upregulated to enhance sulfate uptake from the root and S assimilation (Maruyama-Nakashita et al., 2006). In contrast, key enzymes within different phases of GSL biosynthesis, including MAM1, MAML, CYP79B2/B3, CYP83B1, GST, BCAT and MYB34, are downregulated in *slim1* mutants under S limitation (Maruyama-Nakashita et al., 2006). Moreover, the expression of MYBs regulating aliphatic GSL synthesis in *Arabidopsis* varies under S stress. Under mild S deficient conditions, in the transgenic lines of distinct MYB genes, expression level of MYB28 can be induced, whereas MYB29 and MYB76 expression level were positively correlated with S concentrations (Li et al., 2013). It has been suggested that in *Arabidopsis*, S assimilation is affected by SLIM1, which can be considered as a negative regulator of the R2R3-MYB family of TF, members of which have specific interactions with different components of GSL synthesis pathways (Takahashi et al., 2011; **Figure 6**). qRT-PCR analysis of *Arabidopsis* over-expressed SLIM1 cultured cells confirmed that SLIM1 can suppress the expression of R2R3-MYBs *in vitro* (Frerigmann and Gigolashvili, 2014b). However, the expression level of MYB28, MYB34, MYB51, and MYB122 was neither changed nor increased upon S limitation. To justify this observation, it was suggested that a low substrate (indole or aliphatic GSL) production signal can nullify the negative regulation of MYBs by SLIM1 (Frerigmann and Gigolashvili, 2014b).

Early investigation of S and GSH supply and concentrations in *B. napus* had suggested that under S limitation, GSLs might be degraded within intact plants by myrosinase, without tissue damage (Schnug et al., 1995). In response to S limitation, *Arabidopsis* plants degrade GSLs, so that the released S can be remobilized for production of primary metabolites. In this S recycling process, SLIM1 appears to play an important role, by upregulating the genes encoding enzymes for GSL degradation. This is supported by evidence from transcript analysis of a *Arabidopsis slim1* mutant under S limiting conditions, where a putative thioglucosidase (i.e., myrosinase) were found to be significantly upregulated, to hydrolyze GSLs (Maruyama-Nakashita et al., 2006; **Figure 6**).

Several studies suggest that there is a metabolic association between indole glucosinolates and indole-3-acetic acid (IAA), with GSL being hydrolyzed in consecutive reactions into indole acetonitrile (IAN), which is then hydrolyzed by nitrilase to IAA. Moreover, under S stress hydrolysis of indole GSLs can induce the synthesis of auxin (IAA), enhancing lateral root formation to increase uptake of sulfate from the soil. Although, increased accumulation of IAA has not been reported under S limiting conditions, several investigations suggest that indole GSL hydrolysis does occur under these conditions (Frerigmann and Gigolashvili, 2014b), with associated gene induction of Trp synthesis (Nikiforova et al., 2003), activation of a GSL hydrolysis enzyme (Nikiforova et al., 2003, 2005; Hirai et al., 2004, 2005) and overexpression of nitrilases (Kutz et al., 2002).

MicroRNAs (miRNAs) have a significant regulatory function in controlling gene expression in response to nutrient deficiency, including S deficiency (Sunkar et al., 2007). In *Arabidopsis*, miR395 is involved in the regulation of S assimilation and sulfate transport processes (Bonnet et al., 2004; Jones-Rhoades and Bartel, 2004), by targeting the mRNAs of three ATPS genes involved in S assimilation (Jones-Rhoades and Bartel, 2004; Matthewman et al., 2012; **Figure 6**). Under S starvation, expression of miR395, which can also target the transcripts of SULT2; 1, was found to be upregulated (Allen et al., 2005; Liang and Yu, 2010), which suggests that the rate of S assimilation can also be manipulated by targeting the cell-specific expression pattern of miR395 (Kawashima et al., 2011; Matthewman et al., 2012). Under S deficiency, as opposed to complete S starvation, SLIM1 TF in plants can directly or indirectly induce the expression of miR395 (Kawashima et al., 2009; Liang et al., 2010; **Figure 6**), as can redox signaling (Jagadeeswaran et al., 2014). It has been shown that miR395 is phloem-mobile and can play the role of a long distance signaling molecule in response to changes in plant S status (Buhtz et al., 2010). In addition, miR393, which normally targets auxin receptors, was found to be capable of redirecting the biosynthesis of secondary metabolites from camalexin toward GSLs (Robert-Seilaniantz et al., 2011). To date no such studies have unraveled the role of direct miRNA signaling within the GSL pathway.

The network of interactions outlined above (reviewed in Lewandowska and Sirko, 2008; Koprivova and Kopriva, 2014, 2016; Frerigmann, 2016) suggests that there are significant feedback mechanisms to enable cross-talk between the S and GSL systems (**Figure 6**). This is consistent with earlier studies which demonstrated, through combined metabolomic and transcriptomic analyses, that the major gene families of GSL biosynthesis including MAM, CYP79 and CYP83 were down-regulated under S deficiency (Maruyama-Nakashita et al., 2006). Additional genes encoding SOTs, C-S lyase, GST and AOPs of GSL biosynthesis, have also been found to be down-regulated under S deficiency (Nikiforova et al., 2003, 2005; Hirai et al., 2005; **Figure 6**). This appears to explain the reduction in GSL content typically observed under S starvation. Consequently, to maximize sulfate uptake, assimilation and utilization under S-deficient conditions, plants increase the expression of genes responsible for the S assimilation process (Hirai et al., 2003; Falk et al., 2007).

## A Regulatory Model of GSL Response to S

Overall, there are many lines of evidence to suggest that plant S status has a considerable influence on GSL content. Here, we propose that the interactions between the S and GSL systems act to mediate the distribution of resources from the respective sources and sinks. Moreover, we propose that the specific sets of transporters, signaling molecules and TFs mediate the cross-talk between these sources and sinks in the context of the intracellular, symplastic and vascular systems. Sulfate taken up from soil (primary source) can be assimilated into either primary or secondary metabolites, including GSLs in roots or shoots which can be considered as secondary sources, as well as primary sink tissues. Notably, GSH and Met produced from Cys in the S assimilation process can enter into the GSL biosynthesis pathway within different organelles and tissues. On the other hand, PAPS associated with S assimilation acts as an S donor for sulfation of biologically active GSLs, which can then be transported to the primary (leaf, root and silique) and secondary GSL sink (seed) tissue. As mentioned, SLIM1 can affect miR395, which in turn can regulate uptake, assimilation and transport of sulfate. In order to maintain S homoeostasis, the mi395 signaling molecule provides a feedback mechanism to regulate transporters which then facilitates S assimilation within secondary sources (shoot tissues) (**Figures 4** and **6**) (Liang et al., 2010). As a consequence this mechanism can affect the availability of Cys, GSH, Met, and PAPS for GSL biosynthesis in primary and secondary source and sink tissues (leaf, root, and silique), and can thus limit the accumulation of GSLs in the ultimate sink (seed embryo) at maturity. Moreover, under S deficiency, the TF SLIM1 affects both systems, including S assimilation by SULTR transporters, as well as targeting the key enzymes of GSL biosynthesis (Maruyama-Nakashita et al., 2006). The GSL network is also targeted by a complex network of MYB TFs via SLIM1 as discussed in Section “Molecular Cross-Talk between S Availability and GSL Biosynthesis”. SMIL1 can negatively regulate MYBs under S deficiency, although a signal induced by low indole GSL production can nullify this effect (Li et al., 2013; Frerigmann and Gigolashvili, 2014b).

The overall model (**Figure 6**) provides a working framework to explore the cross-talk between S and GSL systems. However, we would expect there to be additional signaling molecules required to mediate between Cys and GSL chain elongation and core structure synthesis, PAPS and the GSL core structure synthesis, as well as between ATPS and GSL biosynthesis. In *Arabidopsis* there appears to be strong evidence for only six MYBs (MYB 28, 29, 76 for aliphatic and MYB 51, 122, 34 for indole GSL synthesis) being sufficient for signaling the regulation of GSL biosynthesis. However, this may not be the case in brassicas, due to the increased genome complexity, ontogenetic plasticity, and GSL diversity compared with *Arabidopsis*. Additionally, recent identification of four MYC-bHLHs, two new MYBs (MYB115 and MYB118 for aliphatic GSLs synthesis) and a CB5C protein suggests that there may be more distinct or subtle patterns of GSL regulation in *Arabidopsis* under different conditions of S starvation and sufficiency, which needs further investigation. Moreover, there may be further TFs that provide more specific interactions with signaling molecules and sulfate and GSL transporters, as well as with specific biosynthetic steps. Overall, we would expect that under different soil S levels S assimilation rates will vary, which can affect Cys, GSH, PAPS and Met availability and the transcription of genes for GSL biosynthesis.

## Concluding Remarks

As GSLs represent one of the most widely studied classes of plant secondary metabolites, there is an extensive literature describing GSL biosynthesis genes available from the model plant *Arabidopsis and B. oleracea* (Sonderby et al., 2010; Sotelo et al., 2014). Based on this we have developed a model of crop *Brassica* GSL response to S (**Figure 6**). Seeds can be considered as the ultimate sink for GSL accumulation. In order to manipulate the content of GSLs within the seed, our model would suggest that different rates of S provided as fertilizer can make a significant contribution. Also, our model suggests that there is scope to understand the role of tissue-specific GSL biosynthesis genes under different S status.

*Brassica* genomes are three to five times the size of the *Arabidopsis* genome (Paterson et al., 2001) and are more complex, with multiple paralogous gene arising from genome duplication events. This provides considerable scope for gene neo-functionalization in terms of their spatial and temporal regulation (Kroymann, 2011; Mickelbart et al., 2015), and may lead to more sophisticated means of controls for GSL accumulation. Moreover, *Brassica* seeds are more than 10 times larger than those of *Arabidopsis* (Gorsuch et al., 1991), and contain a broader range of GSLs (reviewed in Ishida et al., 2014). Thus at present it is difficult to resolve and understand the complexity and subtlety of interactions in large crop *Brassica* plants based on the available literature from the *Arabidopsis* model, especially where loci associated with GSL response to S have yet to be characterized. Studies with the amphidiploid *Brassica* species are likely to yield additional valuable information to refine the model and establish a better understanding of the molecular regulation of GSL in relation to S availability.

While a number of studies have demonstrated that seed yield and GSL content of *Brassica* crops can increase with S fertilization, any response will ultimately depend on genotype, environment, and existing soil S status. A better understanding of cultivar-specific S requirements to achieve a given seed GSL yield is needed, with appropriate S fertilizer requirements developed according to soil S status. This will be informed by a detailed knowledge of the distribution and remobilization of S and GSLs throughout the development of *Brassica* crops, which will also contribute to completing the framework of our conceptual model (**Figure 6**).

From the evidence we have evaluated, it is clear that the underlying regulatory mechanisms affecting the interaction of S sources and GSL sinks remain to be resolved. As indicated in the model (**Figure 6**), only a few recent studies have identified sulfate transporters, and additionally, only one TF (SLIM1) and one microRNA (miR395) of *Arabidopsis* that are expressed under S deficient conditions. As yet few studies have evaluated the mechanism(s) behind the differential response of plant GSLs in the context of S availability. We are aware that a more complex or sophisticated regulatory framework may exist in the brassicas that integrates the rate of S assimilation, availability of precursors for GSL biosynthesis and seed GSL accumulation in response to different S status.

## Author Contributions

PB carried out the literature review and prepared the manuscript, TR and GK each read, edited, and refined the manuscript.

## Conflict of Interest Statement

The authors declare that the research was conducted in the absence of any commercial or financial relationships that could be construed as a potential conflict of interest.

## Abbreviations

APK: adenosine 5′-phosphosulfate kinase
APS: adenosine 5′-phosphosulfate
ATPS: ATP sulfurylase
Cys: cysteine
GSH: glutathione
GSL: glucosinolate
GST: glutathione *S*-transferease
GTR: glucosinolate transporter
MAM: methylthioalkylmalate
Met: methionine
N: nitrogen
PAPS: 3′-phosphoadenosine 5′-phosphosulfate
qRT-PCR: quantitative real time-polymerase chain reaction
QTL: quantitative trait locus
S: sulfur
SLIM1: Sulfur Limitation 1
SOTs or STs: sulfotransferases
SULTR: sulfate transporter
TF: transcription factor
