# Supplementary material for: Fire and Brimstone: Molecular Interactions between Sulfur and Glucosinolate Biosynthesis in Model and Crop Brassicaceae
Source: Front Plant Sci. 2016 Nov 21;7:1735. doi: 10.3389/fpls.2016.01735 (PMC5116641; doi:10.3389/fpls.2016.01735)
Supplement: Supplementary file 1 [file Table_1.pdf]

Fire and Brimstone: Molecular Interactions between Sulfur and Glucosinolate Biosynthesis in Model and Crop Brassicaceae

Priyakshee Borpatragohain[1], Terry J. Rose [1,2], Graham J. King [1*]

[1]Southern Cross Plant Science, Southern Cross University, Lismore, NSW, Australia.

[2]Southern Cross GeoScience, Southern Cross University, Lismore, NSW, Australia.

Correspondence:

Southern Cross Plant Science, Southern Cross University, Lismore, NSW, 2480, Australia; Tel: (61) 02-6620-3410 Fax: (61) 02-6622-3459

E-mail: graham.king@scu.edu.au

Supplementary Table 1

Effect of S on seed yield, GSL and Met Content. Modified from Falk et al. (2007).

Plant	Organ	Low S treatment	High S treatment	Total GSL at low S	Total GSL at high S	Yield/methionine content at low S	Yield/ methionine content at high S	% change in total GSL	% change in yield	References
**A. Field experiments**										
Brassica oleracea L. *capitata*		0 kg S ha–1	110 kg S ha–1	2.26 µmol gfw–1	2.85 µmol gfw–1	36.1 Mgha-1	39.2 Mgha-1	+ 26	(+) 9	(Rosen et al., 2005)
Brassica oleracea L. *italica*		0 kg S ha–1	23 kg S ha–1	32.1 mg per leaf, 63.6 mg per head	38.0 mg per leaf, 88.3 mg per head	363.4 µg S per mL in leaf 308.6 µg S per mL in head	529.2 µg S per mL in leaf 419.3 µg S per mL in head	+18 leaf (+) 39 head	(+)46 leaf (+) 39 head	(Rangkadilok et al., 2004)
Brassica oleracea L. *italica* var. Marathon		15 kg S ha–1	150 kg S ha–1	51.8 µmol gdw–1 (head, early season), 30.6 µmol gdw–1 (late season)	36.2 µmol gdw–1 (head, early season), 52.5 µmol gdw–1(head, late season)			(–) 30		(Vallejo et al., 2003)

							(+) 72		
Brassica oleracea L. *italica*<br>var. Monterrey	15 kg S ha–1	150 kg S ha–1	32.2 µmol gdw–1(head, early season),<br>84.9 µmol gdw–1(head, late season)	78.4 µmol gdw–1<br>(head, early season),<br>69.6 µmol gdw–1<br>(head, late season)			(+) 144		(Vallejo et al., 2003)
							(–) 18		
Brassica napus L. cv. Bulbul-98	0 kg S ha–1	30 kg S ha–1	13.6 µmol/g	24.6 µmol/g			(+) 11		(Ahmad et al., 2007)
Brassica napus	0 kg S ha–1	80 kg S ha–1	15.8 µmol g–1 seed	41.2 µmol g–1 seed			(+) 161		(Withers and O'Donnell, 1994)
Brassica napus	0 kg S ha–1	100 kg S ha–1	2.59 µmol g–1	4.2 µmol g–1			(+) 62		(Salac et al., 2006)
Brassica napus cv. Dunkeld	0 kg S ha–1	40 kg S ha–1	51.0 µmol g–1	61.67 µmol g–1	1.52 Mg seed ha-1	1.67 Mg seed ha-1	(+) 10.67	(+) 10	(Rehman et al., 2012)

Brassica napus cv. Shiralee	0 kg S ha–1	40 kg S ha–1	56.0 µmol g–1	68.67 µmol g–1	1.54 Mg seed ha-1	1.68 Mg seed ha-1	(+) 12.67	(+) 9	(Rehman et al., 2012)
Brassica juncea canola cv. Arid	0 kg S ha–1	40 kg S ha–1	4.0 µmol g–1 in seed	11.9 µmol g–1 in seed	366 Kg ha-1	1795 Kg ha-1	(+) 198	(+) 390	(Malhi et al., 2007)
Brassica juncea canola cv. Amulet	0 kg S ha–1	40 kg S ha–1	12.6 µmol g–1 in seed	20.7 µmol g–1 in seed	1045 Kg ha-1	1870 Kg ha-1	(+) 64	(+) 79	(Malhi et al., 2007)
Brassica juncea mustard cv. Cutlass	0 kg S ha–1	40 kg S ha–1	61.0 µmol g–1 in seed	98.6 µmol g–1 in seed	1090 Kg ha-1	2325 Kg ha-1	(+) 62	(+) 113	(Malhi et al., 2007)
Brassica napus cv InVigor 2663 hybrid canola	0 kg S ha–1	40 kg S ha–1	2.4 µmol g–1 in seed	7.9 µmol g–1 in seed	874 Kg ha-1	3093 Kg ha-1	(+) 229	(+) 254	(Malhi et al., 2007)
Brassica napus cv Lisek	0 kg S ha–1	90 kg S ha–1	9.29 µmol g–1 in seed	11.55 µmol g–1 in seed					(Jankowski et al., 2008)
**B. Greenhouse experiments**									

Brassica napus	0.9 mM SO_4^{2-}	18.3 mM	11.3 µmol gdw–1	153.8 µmol gdw–1	32 g plant-1 (seed yield) 6.93 mg gdw-1 methionine	48 g plant-1 (seed yield) 8.47 mg gdw-1 methionine	(+) 1261	(+) 50 yield (+) 22 methionine	(Davik and Bakken, 1999)
Brassica rapa	0.5 mM SO_4^{2-}	1.0 mM	28.4 µmol gdw–1	80 µmol gdw–1			(+) 182		(Kim et al., 2002)
**C. Hydroponics/ tissue culture experiments**									
Brassica oleracea L. Acephala	0.125 mM SO_4^{2-}	2.0 mM SO_4^{2-}	0.59 µg gdw–1	3.97 µg gdw–1			(+) 573		(Kopsell et al., 2003)
Brassica napus	0 mM SO_4^{2-}	1.0 mM SO_4^{2-}	8.14 µmol gdw–1	122.1 µmol gdw–1			(+) 1400		(Blake-Kalff et al., 1998)
Brassica oleracea L. *italica*	0 mg L–1 SO_4^{2-}	29.2 mg L–1 SO_4^{2-}	56.1 µmol gdw–1	33.8 µmol gdw–1			(–) 40		(Aires et al., 2006)
Brassica napus var. Bienvenu	0 mM SO_4^{2-}	0.5 mM SO_4^{2-}	100 µg kg fresh wt–1	4669 µg kg fresh wt–1			(+) 4569		(Dubuis et al., 2005)

Brassica oleracea L. *gemmifera*	0 ppm SO_4^{2-}	15.0ppm SO_4^{2-}	110 µmol gdw–1	168 µmol gdw–1 (not infested with aphids)	(+) 53	(Yusuf and Collins, 1998)
			90 µmol gdw–1	66 µmol gdw–1 (infested with aphids)	(–) 27	

REFERENCES

Ahmad, G., Jan, A., Arif, M., Jan, M.T., and Khattak, R.A. (2007). Influence of nitrogen and sulfur fertilization on quality of canola (*Brassica napus* L.) under rainfed conditions. *Journal of Zhejiang University. Science. B* 8**,** 731-737. doi: 10.1631/jzus.2007.B0731.

Aires, A., Rosa, E., and Carvalho, R. (2006). Effect of nitrogen and sulfur fertilization on glucosinolates in the leaves and roots of broccoli sprouts (*Brassica oleracea* var. italica). *Journal of the Science of Food and Agriculture* 86**,** 1512-1516.

Blake-Kalff, M.M.A., Harrison, K.R., Hawkesford, M.J., Zhao, F.J., and McGrath, S.P. (1998). Distribution of Sulfur within Oilseed Rape Leaves in Response to Sulfur Deficiency during Vegetative Growth. *Plant Physiology* 118**,** 1337-1344. doi: 10.1104/pp.118.4.1337.

Davik, J., and Bakken, A. (1999). Seed yield and sulphur partitioning in two inbred lines of low and high glucosinolate oilseed rape (*Brassica napus* L.) and their hybrids at three levels of sulphur supply. *Acta Agric. Sc and. Sect. B. Soil and Plant Sci* 49**,** 184-188.

Dubuis, P.H., Marazzi, C., Städler, E., and Mauch, F. (2005). Sulphur deficiency causes a reduction in antimicrobial potential and leads to increased disease susceptibility of oilseed rape. *Journal of Phytopathology* 153**,** 27-36.

Jankowski, K., Budzyñski, W., and Szymanowski, A. (2008). Effect of sulfur on the quality of winter rape seeds. *Journal of Elementology* 13**,** 521-534.

Kim, S.-J., Matsuo, T., Watanabe, M., and Watanabe, Y. (2002). Effect of nitrogen and sulphur application on the glucosinolate content in vegetable turnip rape (*Brassica rapa* L.). *Soil Science and Plant Nutrition* 48**,** 43-49. doi: 10.1080/00380768.2002.10409169.

Kopsell, D.E., Kopsell, D.A., Randle, W.M., Coolong, T.W., Sams, C.E., and Curran-Celentano, J. (2003). Kale carotenoids remain stable while flavor compounds respond to changes in sulfur fertility. *Journal of agricultural and food chemistry* 51**,** 5319-5325.

Malhi, S., Gan, Y., and Raney, J. (2007). Yield, Seed Quality, and Sulfur Uptake of Oilseed Crops in Response to Sulfur Fertilization. *Agronomy Journal* 99**,** 570-577.

Rangkadilok, N., Nicolas, M.E., Bennett, R.N., Eagling, D.R., Premier, R.R., and Taylor, P.W. (2004). The effect of sulfur fertilizer on glucoraphanin levels in broccoli (*B. oleracea* L. var. italica) at different growth stages. *Journal of agricultural and food chemistry* 52**,** 2632-2639.

Rehman, H.u., Farooq, M., Iqbal, Q., Wahid, A., Basra, S.M.A., and Afzal, I. (2012). Sulphur application improves the growth, seed yield and oil quality of canola. *Acta Physiol Plant*. doi: 10.1007/s11738-013-1331-9.

Rosen, C., Fritz, V., Gardner, G., Hecht, S., Carmella, S., and Kenney, P. (2005). Cabbage yield and glucosinolate concentrations as affected by nitrogen and sulfur fertility. *HortScience* 40**,** 1493-1498.

Salac, I., Haneklaus, S., Bloem, E., Booth, E., Sutherland, K., Walker, K., et al. (2006). Influence of sulfur fertilization on sulfur metabolites, disease incidence and severity of fungal pathogens in oilseed rape in Scotland. *Landbauforschung Vôlkenrode* 56**,** 1-4.

Vallejo, F., Tomás-Barberán, F.A., Benavente-García, A.G., and García-Viguera, C. (2003). Total and individual glucosinolate contents in inflorescences of eight broccoli cultivars grown under various climatic and fertilisation conditions. *Journal of the Science of Food and Agriculture* 83**,** 307-313.

Withers, P.J., and O'Donnell, F.M. (1994). The response of double-low winter oilseed rape to fertiliser sulphur. *Journal of the Science of Food and Agriculture* 66(1)**,** 93-101.

Yusuf, S.W., and Collins, G.G. (1998). Effect of soil sulphur levels on feeding preference of Brevicoryne brassicae on Brussels sprouts. *Journal of Chemical Ecology* 24(3)**,** 417-424.
