# Supplementary material for: Fire and Brimstone: Molecular Interactions between Sulfur and Glucosinolate Biosynthesis in Model and Crop Brassicaceae
Source: Front Plant Sci. 2016 Nov 21;7:1735. doi: 10.3389/fpls.2016.01735 (PMC5116641; doi:10.3389/fpls.2016.01735)
Supplement: Supplementary file 2 [file Table_2.pdf]

# Fire and Brimstone: Molecular Interactions between Sulfur and Glucosinolate Biosynthesis in Model and Crop Brassicaceae

Priyakshee Borpatragohain[1], Terry J. Rose [1,2], Graham J. King [1*]

[1]Southern Cross Plant Science, Southern Cross University, Lismore, NSW, Australia.

[2]Southern Cross GeoScience, Southern Cross University, Lismore, NSW, Australia.

**Correspondence:**

Southern Cross Plant Science, Southern Cross University, Lismore, NSW, 2480, Australia; Tel: (61) 02-6620-3410 Fax: (61) 02-6622-3459

E-mail: graham.king@scu.edu.au

## Supplementary Table 2

Candidate genes for GSLs synthesis and signalling in annotated brassica genomes. Summarised from Wang et al. (2011); Chalhoub et al. (2014); Liu et al. (2014)

| Group name | AGI | B.rapa ID (Ar) | B.rapa ID (Ar) ro18 | Blocks | specific chromosome of B.rapa | B.oleracea ID (Co) | B.napus (An sbugenome) ID | B.napus (Cn sbugenome) ID | Missing gene detailed analysis | status *B. napus* (after final validations) |
|---|---|---|---|---|---|---|---|---|---|---|
| **Transcription factors** | | | | | | | | | | |
| **Dof1.1** | At1g07640 | Bra031588 | Bra031960-RA | A | (A09) | Bo8g112940 | | | | |
| | | Bra030696 | Bra005208-RA | A | (A08) | Bo8g010700 | BnaA08g29080D | [NS]chrA08:18942871-18943761 | Cn missing | not validated as lost |
| | | | | | | Bo5g008360 | | | | |
| **IQD1-1** | At3g09710 | Bra034081 | Bra017665-RA | F | (A01) | Bo1g144340 | BnaA01g32120D | BnaC01g39030D | | |
| | | Bra001299 | Bra008263-RA | F | (A03) | Bo3g061890 | BnaA03g30550D | BnaC03g35860D | | |
| **MYB28** | At5g61420 | Bra012961 | Bra025143-RA | X | (A03) | Bo7g098590 | BnaA03g40190D | [NS]chrCnn_random:42331702-42332506 | Inside no syntenic model [NS] random | |

| | | | | | | | | | | |
|---|---|---|---|---|---|---|---|---|---|---|
| | | Bra035929 | Bra000631-RA | X | (A09) | Bo9g014610 | [NS]chrC09:3099350-3100161 | BnaC09g05300D | An missing | LOST An |
| | | Bra029311 | Bra009052-RA | X | (A02) | Bo2g161590 | [S]chrAnn_random:6899080-6899225 | [NS]chrAnn_random:6898533-6898665 | | HE: 5DAn2+/5DCn2- |
| **MYB29** | At5g07690 | Bra009245 | Bra034417-RA | R | (A10) | Bo9g175680 | | | | |
| | | Bra005949 | Bra005701-RA | R | (A03) | Bo3g004500 | BnaA03g02170D | BnaC03g03210D | | |
| **MYB76** | At5g07700 | | | | | | | | | |
| **MYB34** | At5g60890 | Bra013000 | Bra009027-RA | X | (A03) | Bo7g098110 | BnaA03g39790D | BnaCnng21270D | | |
| | | Bra035954 | Bra000610-RA | X | (A09) | Bo9g014380 | BnaA09g05480D | BnaC09g05060D | | |
| | | Bra029350 | | X | (A02) | Bo2g161170 | BnaAnng06630D | BnaC02g41860D | | |
| | | Bra029349 | | X | (A02) | Bo2g161180 | BnaAnng06640D | BnaC02g41860D | | |
| **MYB51** | At1g18570 | Bra025666 | Bra031655-RA | A | (A06) | Bo5g025570 | BnaA06g12690D | BnaC05g14250D | | |
| | | Bra031035 | Bra023576-RA | A | (A09) | Bo8g104300 | BnaA09g44500D | BnaC08g37160D | | |

| | | | | | | | | |
|---|---|---|---|---|---|---|---|---|
| | | Bra016553 | Bra021185-RA | A | (A08) | Bo8g067910 | BnaA08g22580D | BnaC08g18090D |
| | | | Bra011642-RA | | | | | |
| | | | Bra004739-RA | | | | | |
| **MYB122** | At1g74080 | Bra015939 | | E | (A07) | Bo6g008480 | BnaA07g31220D | BnaC06g34910D |
| | | Bra008131 | | E | (A02) | Bo2g080900 | BnaA02g16690D | BnaC02g22630D |
| **Side-chain elongation** | | | | | | | | |
| **BCAT-4** | At3g19710 | Bra022448 | Bra029343-RA | F | (A05) | Bo5g113720 | BnaA05g36800D | BnaC05g33030D |
| | | Bra001761 | Bra027923-RA | F | (A03) | Bo3g073430 | BnaA03g35400D | BnaC03g41100D |
| | | | Bra023142-RA | | | | | |
| | | | Bra010713-RA | | | | | |
| | | | Bra008649-RA | | | | | |
| **BAT5** | At4g12030 | Bra029434 | Bra009534-RA | P | (A09) | Bo9g094080 | BnaA09g21170D | BnaC09g23550D |

| | | | | | | | | |
|---|---|---|---|---|---|---|---|---|
| | | Bra000760 | Bra007801-RA | P | (A03) | Bo3g045530 | BnaA03g24950D | BnaC03g73000D |
| | | Bra019352 | Bra002450-RA | | | | | |
| **MAM1** | At5g23010 | Bra013007 | Bra036842-RA | Q | (A03) | Bo7g098000 | BnaA03g39720D | BnaCnng21190D |
| | | Bra029355 | Bra025109-RA | Q | (A02) | Bo2g161100 | BnaA02g33040D | BnaC02g41790D |
| | | Bra018524* | Bra024037-RA | O | (A02) | Bo2g102060 | BnaA02g20830D | BnaC02g26810D |
| **MAM3** | At5g23020 | Bra013009 | Bra009023-RA | Q | (A03) | Bo7g098000 | BnaA03g39710D | BnaCnng21190D |
| | | Bra013011 | Bra009021-RA | Q | (A03) | Bo7g098000 | BnaA03g39680D | BnaCnng21190D |
| | | Bra029356 | | Q | (A02) | Bo2g161100 | BnaA02g33040D | BnaC02g41790D |
| | | Bra021947* | | J | (A04) | Bo4g183080 | BnaA04g20510D | [NS]chrA03:19821938-19822196 |
| **IPMI LSU1** | At4g13430 | Bra032708 | Bra035540-RA | T | (A04) | Bo4g131050 | BnaA04g06890D | BnaCnng15040D |
| | | Bra040341 | Bra029915-RA | T | (A08) | Bo00805s020 | | |

| | | | | | | | | | |
|---|---|---|---|---|---|---|---|---|---|
| **IPMI SSU2** | At2g43100 | Bra004744 | Bra007416-RA | J | (A05) | Bo4g018590 | [NS]chrA05_random:134636-135412 | BnaC04g52970D | |
| **IPMI SSU3** | At3g58990 | Bra004743 | | | | Bo4g018580.1 | | | |
| | | | | | | Bo3g036360.1 | | | |
| **IPMDH1** | At5g14200 | Bra023450 | | R | (A02) | Bo2g011730 | BnaA02g02020D | [NS]chrCnn_random:64596419-64596639 | inside-no_syntenic_model-[NS]-random |
| **IPMDH3** | At1g31180 | | | | | | | | |
| **BCAT-3** | At3g49680 | Bra017964 | | M | (A06) | Bo8g078930 | BnaA06g15650D | [NS]chrCnn_random:25107841-25108074 | inside-no_syntenic_model-[NS]-random |
| | | Bra029966 | | M | (A01) | Bo1g080200 | BnaA01g20890D | [S]chrC01:22970928-22971059 | inside-no_syntenic_model-[S]-complete |
| **Core structure formation** | | | | | | | | | |
| **CYP79F1** | At1g16410 | Bra026058 | | A | (A06) | Bo5g021810 | BnaA06g11010D | BnaC05g12520D | |
| **CYP79F2** | At1g16400 | | | | | | | | |

| | | | | | | | | |
|---|---|---|---|---|---|---|---|---|
| **CYP79A2** | At5g05260 | Bra009100 | R | (A10) | Bo9g177260 | BnaA10g25130D | BnaC09g50060D | |
| | | Bra028764 | R | (A02) | Bo2g006900 | BnaAnng01260D | BnaC02g02390D | |
| **CYP79B2** | At4g39950 | Bra011821 | U | (A01) | Bo1g002970 | BnaA01g34610D | BnaC01g00800D | |
| | | Bra010644 | U | (A08) | Bo3g152800 | BnaA08g16100D | BnaC03g60820D | |
| | | Bra017871* | U | (A03) | Bo7g118840 | [S]chrA03:29024090-29025083 | [NS]chrC07_random:2929352-2930345 | Inside no syntenic model [S] complete |
| **CYP79B3** | At2g22330 | Bra030246 | I | (A04) | Bo4g149550 | BnaA04g12790D | [S]chrC04:36225448-36226452 | Inside no syntenic model [S] complete |
| **CYP83A1** | At4g13770 | Bra032734 | T | (A04) | Bo4g130780 | BnaA04g06630D | BnaC04g29320D | |
| | | Bra016908* | J | (A04) | Bo4g191120 | BnaA04g24160D | BnaC04g47910D | |
| **CYP83B1** | At4g31500 | Bra034941* | R | (A08) | Bo8g024390 | BnaA08g04520D | BnaC08g05690D | |

| | | | | | | | | | |
|---|---|---|---|---|---|---|---|---|---|
| **GSTF9** | At2g30860 | Bra021673 | IJ | (A04) | Bo4g173610 | BnaA04g17910D | BnaC04g41510D | | |
| | | Bra022815 | IJ | (A03) | Bo3g024840 | BnaA03g14140D | BnaC03g17110D | | |
| **GSTF10** | At2g30870 | Bra022816 | IJ | (A03) | Bo3g024850 | BnaA03g14150D | BnaC03g17120D | | |
| **GSTF11** | At3g03190 | Bra032010 | F | (A05) | Bo5g150180 | BnaA05g32420D | BnaCnng06300D | | |
| **GSTU20** | At1g78370 | Bra003645* | E | (A07) | Bo6g045200 | BnaA07g20570D | BnaC06g20430D | | |
| **GGP1** | At4g30530 | Bra011201 | U | (A01) | Bo1g012070 | BnaA01g06540D | BnaC01g07810D | | |
| | | Bra024068 | U | (A03) | Bo7g114570 | BnaA03g50240D | BnaC07g42720D | | |
| | | Bra010282 | U | (A08) | | | | | |
| | | Bra010283 | U | (A08) | Bo3g175530 | BnaA08g13020D | [NS]chrA08:11366369-11366700 | Cn missing | not validated as lost |
| **SUR1** | At2g20610 | Bra036490 | H | (A07) | Bo7g003330 | BnaA07g00460D | [S]chrC07:490537-490603 | Inside no syntenic model [S] complete | |
| | | Bra036703 | H | (A09) | | | | | |

| | | | | | | | | |
|---|---|---|---|---|---|---|---|---|
| **UGT74B1** | At1g24100 | Bra024634 | B | (A09) | Bo5g041080 | BnaA09g29790D | [NS]chrC05_random:1636643-1637374 | Inside no syntenic model [NS] random |
| **UGT74C1** | At2g31790 | Bra005641 | J | (A05) | Bo4g049480 | BnaA05g11170D | BnaC04g12860D | |
| | | Bra021743 | J | (A04) | Bo4g177540 | BnaA04g18440D | BnaC04g42530D | |
| **ST5a** | At1g74100 | Bra015935 | E | (A07) | Bo6g008450 | BnaA07g31260D | BnaC06g35000D | |
| | | Bra008132 | E | (A02) | Bo2g080910 | [NS]chrAnn_random:31132802-31133821 | BnaC02g22640D | |
| **ST5b** | At1g74090 | Bra015938 | E | (A07) | Bo6g008470 | BnaA07g31230D | BnaC06g34930D | |
| | | Bra015936 | E | (A07) | Bo6g008460 | BnaA07g31250D | BnaC06g34970D | |
| | | Bra003817 | E | (A07) | | | | |
| | | Bra003818 | E | (A07) | | | | |
| | | Bra003726 | E | (A07) | | | | |

| | | | | | | | |
|---|---|---|---|---|---|---|---|
| | | Bra027880* | D | (A09) | | | |
| | | Bra027117* | D | (A09) | | | |
| | | Bra027118* | D | (A09) | | | |
| | | Bra027623* | D | (A09) | | | |
| | | Bra031476* | D | (A01) | | | |
| **ST5c** | At1g18590 | Bra025668 | A | (A06) | Bo5g025610 | BnaA06g12720D | BnaC05g14270D |
| **Secondary modification** | | | | | | | |
| **FMOGS-OX1** | At1g65860 | | | | | | |
| **FMOGS-OX2** | At1g62540 | Bra027035* | D | (A09) | Bo9g037180 | BnaA09g13190D | BnaC09g13360D |
| **FMOGS-OX3** | At1g62560 | | | | | | |
| **FMOGS-OX4** | At1g62570 | | | | | | |
| **FMOGS-OX5** | At1g12140 | Bra026988 | A | (A09) | Bo8g108390 | BnaA09g47360D | BnaC08g41540D |

| | | | | | | | |
|---|---|---|---|---|---|---|---|
| | | Bra016787 | A | (A08) | Bo8g062610 | BnaA08g24880D | BnaC08g15390D |
| **AOP1** | At4g03070 | Bra034182 | O | (A09) | | | |
| | | Bra034181 | O | (A09) | | | |
| | | Bra000847 | O | (A03) | | | |
| **AOP2** | At4g03060 | Bra034180 | O | (A09) | Bo9g006240 | BnaA09g01260D | BnaC09g00410D |
| | | Bra000848 | O | (A03) | Bo3g052110 | BnaA03g25870D | BnaC03g30450D |
| | | Bra018521 | O | (A02) | Bo2g102190 | BnaA02g20860D | BnaC02g26740D |
| **AOP3** | At4g03050 | | | | | | |
| **GSL-OH** | At2g25450 | Bra021670* | IJ | (A04) | Bo4g173530 | BnaA04g17890D | BnaC04g41490D |
| | | Bra021671* | IJ | (A04) | Bo4g173560 | BnaA04g17890D | BnaC04g41490D |
| | | Bra022920* | J | (A03) | | | |
| **CYP81F2** | At5g57220 | Bra002747 | W | (A10) | Bo9g131960 | BnaA10g11290D | BnaC09g32980D |

| | | | | | | | | |
|---|---|---|---|---|---|---|---|---|
| | | Bra020459 | W | (A02) | Bo2g032590 | BnaA02g08270D | BnaC02g11750D | |
| | | Bra006830 | W | (A03) | Bo3g019420 | BnaA03g10380D | BnaC03g13070D | |
| **Co-substrate pathways** | | | | | | | | |
| **BZO1p1** | At1g65880 | Bra004132 | E | (A07) | Bo6g027260 | BnaA07g25490D | BnaC06g27280D | |
| | | Bra039743 | E | (A02) | | | | |
| **APK1** | At2g14750 | Bra013120 | GH | (A03) | Bo3g088400 | BnaA03g38670D | [NS]chrC09:5568420-5568717 | Inside no syntenic model [NS] real ns |
| | | Bra039818 | GH | (S178) | Bo9g022580 | BnaA09g08410D | BnaC09g08710D | |
| **APK2** | At4g39940 | Bra011822 | U | (A01) | Bo1g002960 | BnaA01g34620D | BnaC01g00790D | |
| | | Bra010645 | U | (A08) | Bo3g152770 | BnaA08g16110D | BnaC03g60800D | |
| | | Bra017872* | U | (A03) | Bo7g118850 | BnaA03g54400D | [NS]chrC07_random:2934264-2934567 | Inside no syntenic model [NS] random |

| | | | | | | | | |
|---|---|---|---|---|---|---|---|---|
| **GSH1/PAD2** | At4g23100 | Bra013675 | U | (A01) | Bo1g033120 | BnaA01g12900D | [NS]chrC01_random:635625-635927 | Inside no syntenic model [NS] random |
| | | Bra019333 | U | (A03) | Bo7g108480 | BnaA03g45730D | BnaC07g37900D | |
| | | Bra019332 | U | (A03) | Bo7g108500 | BnaA03g45730D | BnaC07g37950D | |
| **CHY1** | At5g65940 | Bra031802 | X | (A02) | Bo2g167570 | BnaA02g34880D | BnaC02g45100D | |
| | | Bra039968* | IJ | (A04) | | | | |
| | | Bra039975* | IJ | (A04) | | | | |
| | | Bra018392* | IJ | (A05) | | | | |
| **AAO4** | At1g04580 | Bra015330 | A | (A10) | Bo5g004710 | BnaA10g02720D | BnaC05g02700D | |
| | | Bra011762 | | | Bo1g003710 | BnaA01g00510D | BnaC01g01500D | |
| | | Bra011761 | | | Bo1g003710 | BnaA01g00520D | BnaC01g01500D | |

| | | | | |
|---|---|---|---|---|
| Bra011759 | Bo1g004730 | BnaA01g00520D | BnaC01g01530D | |
| Bra011758 | Bo1g004740 | BnaA01g00570D | BnaC01g01540D | |
| Bra023629 | Bo2g013840 | [NS]chrAnn_random:16922504-16923140 | [S]chrC02:3990264-3990915 | |
| Bra006417 | Bo3g012730 | BnaA03g06690D | BnaC03g08560D | |
| Bra022816 | Bo3g024850 | BnaA03g14150D | BnaC03g17120D | |
| Bra004743 | Bo4g018580 | [NS]chrA05_random:130347-131105 | BnaC04g52960D | Inside no syntenic model [NS] random |
| Bra012270 | Bo7g059680 | BnaA07g11060D | BnaC07g14650D | |
| Bra012269 | Bo7g059670 | BnaA07g11070D | BnaC07g14640D | |
| Bra012268 | Bo7g059680 | BnaA07g11080D | BnaC07g14650D | |
| Bra013958 | Bo9g159960 | BnaA10g17060D | BnaC09g40240D | |
| Bra010597 | Bo3g153480 | BnaA08g15650D | BnaC03g61420D | |

| | | | | |
|---|---|---|---|---|
| Bra010598 | | Bo3g153450 | BnaA08g15660D | BnaC03g61420D |
| Bra016432 | | Bo8g070660 | BnaA08g21450D | BnaC08g19690D |
| Bra016433 | | Bo8g070650 | BnaA08g21450D | BnaC08g19680D |

## REFERENCES

Chalhoub, B., Denoeud, F., Liu, S., Parkin, I.A.P., Tang, H., Wang, X., et al. (2014). Early allopolyploid evolution in the post-Neolithic *Brassica napus* oilseed genome. *Science* 345**,** 950-953. doi: 10.1126/science.1253435.

Liu, S., Liu, Y., Yang, X., Tong, C., Edwards, D., Parkin, I.A., et al. (2014). The *Brassica oleracea* genome reveals the asymmetrical evolution of polyploid genomes. *Nat Commun* 5**,** 3930. doi: 10.1038/ncomms4930.

Wang, H., Wu, J., Sun, S., Liu, B., Cheng, F., Sun, R., et al. (2011). Glucosinolate biosynthetic genes in *Brassica rapa*. *Gene* 487**,** 135-142. doi: 10.1016/j.gene.2011.07.021.
