# Supplementary material for: Fire and Brimstone: Molecular Interactions between Sulfur and Glucosinolate Biosynthesis in Model and Crop Brassicaceae
Source: Front Plant Sci. 2016 Nov 21;7:1735. doi: 10.3389/fpls.2016.01735 (PMC5116641; doi:10.3389/fpls.2016.01735)
Supplement: Supplementary file 3 [file Table_3.pdf]

Fire and Brimstone: Molecular Interactions between Sulfur and Glucosinolate Biosynthesis in Model and Crop Brassicaceae

Priyakshee Borpatragohain[1], Terry J. Rose [1,2], Graham J. King [1*]

[1]Southern Cross Plant Science, Southern Cross University, Lismore, NSW, Australia.

[2]Southern Cross GeoScience, Southern Cross University, Lismore, NSW, Australia.

Correspondence:

Southern Cross Plant Science, Southern Cross University, Lismore, NSW, 2480, Australia; Tel: (61) 02-6620-3410 Fax: (61) 02-6622-3459

E-mail: graham.king@scu.edu.au

Supplementary Table 3

Theoretical calculation of S in sink (GSL and proteins) component as percent of seed mass

**Molecule**	**MW**	Number of Methionine (M) and Cystein (C)	**Total no. of S atoms**	**S MW**	**Proportion of molecule as S**	**Proportion of Molecule per g seed**	**S-sink (sinigrin or protein) µmol/g of seed**	**S µM /g of seed**	**S per g seed in sink component**	**S in sink component as % of seed mass**
GSLs (Sinigrin)	397.46		2	64	0.16	0.04 – 0.05	100-130 (maximum GSLs content in brassicas)	3.97 – 6.72	0.0064 – 0.00832	**0.006 – 0.008%**
Cruciferins	400,000	M=4, C= 5	9	288	0.0007	0.15	2.5	0.38	0.0001	**0.000%**
Napins	9,000	M=5, C=8	13	416	0.05	0.15	111.11	16.67	0.0069	**0.007%**
Seed protein total						0.300			0.007	**0.007%**

REFERENCES

Bhandari, S.R., Jung Su, J., and Jun Gu, L. (2015). Comparison of Glucosinolate Profiles in Different Tissues of Nine Brassica Crops. *Molecules* 20**,** 15827-15841. doi: 10.3390/molecules200915827.

Rangkadilok, N., Nicolas, M.E., Bennett, R.N., Premier, R.R., Eagling, D.R., and Taylor, P.W. (2002). Determination of sinigrin and glucoraphanin in *Brassica* species using a simple extraction method combined with ion-pair HPLC analysis. *Scientia Horticulturae* 96, 27-41.
